# Pragmatic Micrometre to Millimetre Calibration Using Multiple Methods for Low-Coherence Interferometer in Embedded Metrology Applications

**DOI:** 10.3390/s21155101

**Published:** 2021-07-28

**Authors:** Tom Hovell, Jon Petzing, Laura Justham, Peter Kinnell

**Affiliations:** Wolfson School of Mechanical, Electrical and Manufacturing Engineering, Loughborough University, Loughborough LE11 3TU, UK; J.Petzing@lboro.ac.uk (J.P.); L.Justham@lboro.ac.uk (L.J.); P.Kinnell@lboro.ac.uk (P.K.)

**Keywords:** calibration, metrology, low-coherence interferometry

## Abstract

In-situ metrology utilised for surface topography, texture and form analysis along with quality control processes requires a high-level of reliability. Hence, a traceable method for calibrating the measurement system’s transfer function is required at regular intervals. This paper compares three methods of dimensional calibration for a spectral domain low coherence interferometer using a reference laser interferometer versus two types of single material measure. Additionally, the impact of dataset sparsity is shown along with the effect of using a singular calibration dataset for system performance when operating across different media.

## 1. Introduction

On-machine metrology is a growing requirement for many high-value manufacturing processes, ensuring tolerances of complex geometric parts are adhered to, and for process control. This is being pushed in part by an increase in components dimensionally constrained in terms of geometric form and surface texture, requiring dimensional information on the part, spanning orders of magnitude [1].

Implementation of in-situ dimensional sensing into the machining environment has been historically difficult due to the hostile operating conditions. However, low coherence interferometry (LCI) is a proven measurement technique that has shown promise for operating in dynamic environments, with a small footprint due to straight forward integration into fibre optic systems and has been shown to effectively work in various media [2,3,4].

To ensure that precise and accurate measurements are produced from such embedded sensors, there is a requirement to investigate how these instruments might be calibrated whilst in-situ for multiscale (micrometric to millimetric) measurements to ensure measurement traceability. This includes to what extent the relationship between sensor and the measurand needs to be measured for accurate representation, and how the sensor response changes due to operating in various environments or media.

The use of step heights has been extensively used for measurement tool calibration [5], with gauge blocks being one of the most common artefacts [6] due to their range of lengths, materials and availability [7]. Three-dimensional artefacts for areal calibration have also been developed for the determination of surface characteristics and lateral resolution [8,9]. Alternatively, transparent artefacts for optical micro-CMM [10] and optical coherence tomography (OCT) [11,12,13] are also present in the literature.

The calibration of sensor operating characteristics should be completed at regular intervals as determined by the measurement context, hence such a calibration regime should be efficient, robust, inexpensive and simple to implement. The literature has shown that such a regime can potentially be achieved through the use of multiple step heights [14] and across a user defined measurement volume [15]. However, the introduction of an additional scanning axis into the calibration procedure increases the impact of the mechanical system on the measurement and increases the time required for calibration.

Calibration on-machine should be a timely process to reduce machine downtime. Thus, the ability to limit the amount of required datapoints along the measurement axis for an acceptable level of residual error is beneficial and would allow for the practical selection of reference media dimensions.

The work presented here investigates the calibration of a spectral domain fibre deployed LCI sensor via three distinct methodologies: the use of a laser interferometer (Renishaw XL-80) as a traceable reference versus the creation of an absolute scale reference through the use of calibrated reference materials, either a step height or a glass coverslip. We demonstrate the use of slip gauges to create a step height calibrated by measurement on a Bruker NPFLEX, a reference scale is created using the calibrated step as a traceable two point measurement at discrete locations across the operating range of the LCI sensor [15]. We also demonstrate the use of a glass coverslip as a reference material, taking advantage of LCI tomographic measurement ability, removing the requirement for a lateral scanning procedure as required with the step height. The impact of calibration dataset sparsity on the overall quality of the calibration curve is investigated by varying the datapoint density on the interferometer calibration datasets whilst observing the impact on residual error across the entire measurement span. Additionally, sensor performance across the operational range in air, paraffin oil, water and metal working fluid (MWF), along with the potential for translating calibration datasets for operation in other media, is explored. Investigation into the impact of various liquid media on the sensor performance is important if integration into the manufacturing environment is to occur. These liquids were selected as they are commonly used as dielectric mediums for processes such as electro discharge machining and emulsifiers for lubrication or for flushing debris.

## 2. Method

### 2.1. Experimental Setup

**Figure 1:**
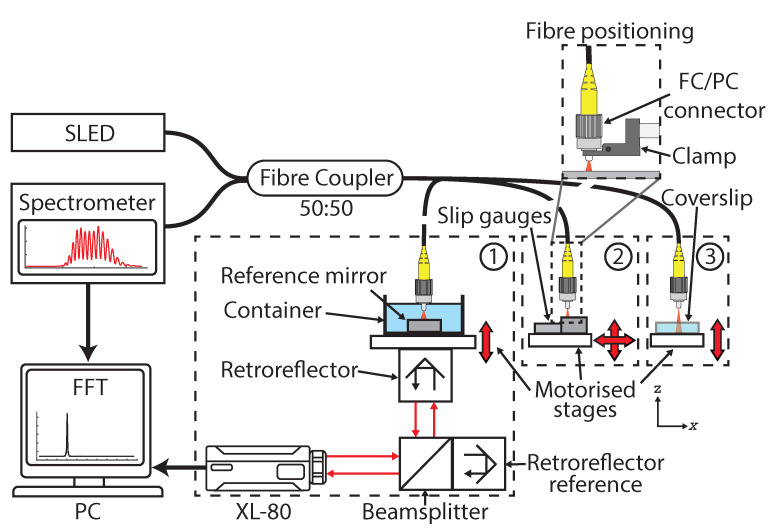
Experimental setup demonstrating all three calibration routines. (**1**) Calibration via Renishaw XL-80; (**2**) calibration via scanning across a calibrated step-height formed from slip gauges; and (**3**) calibration using a calibrated glass coverslip as a reference material.

The optomechanical experimental setup in Figure 1 shows the LCI system in the three calibration configurations covered within this paper. The LCI sensor used here has been presented in previous work [2]; it is a fully fibre-enclosed based implementation with a common path, which is used for both the reference and sample signal, taking advantage of reduced sensitivity to vibrations, thermal fluctuations and humidity, and removing the requirement for dispersion compensation between signals [16,17]. A spectral or Fourier-domain approach is implemented due to the advantages of high acquisition speed and sensitivity over time-domain methods [18]. The system consists of a superluminescent diode (EXS210068-01, Beratron, 850 nm) with a 3-dB bandwidth of 58 nm and an emitting power of 5.14 mW at 160 mA, a single-mode fibre coupler with a splitting ratio of 50:50 for beam splitting and coupling. The spectrometer used (MayaPro2000, Ocean Optics) has an operating speed of 125 Hz with a 2048 × 64 pixel array, a starting wavelength of 756 nm and a spectral range of 174 nm with a resolution of 0.21 nm, giving a theoretical axial operating range of approximately 2.1 mm before aliasing.

A fibre clamp was used to hold the end of the single-mode fibre (780HP with FC/PC connector) perpendicular to the object target. The theory and data processing behind the LCI sensor measurements are covered in previous works [2,4]. To perform scanning measurements, a set of 3-axis stepper motor driven translation stages (MFA-PPD, Newport) in an XYZ configuration were used. The stages have a minimum incremental motion of 0.1 μm in each axis with a manufacturer stated accuracy of ±0.9 μm and a typical bi-directional repeatability of ±0.2 μm, and were driven by a 3-axis motion controller and driver (ESP 301, Newport) controlled by a custom-made LabVIEW program V2019. In this work, the stages are used for positioning the sample and are not used as a reference in the calibration procedure. Hence, it is only required that the linear positioning is sufficient to obtain readings at the required resolution along the translation axis. However, it is fully anticipated that there are stage related imperfections, Newport quotes uncertainties in pitch ± 25 μrad, compliance in pitch 80 μrad/Nm, yaw ± 30 μrad, compliance in yaw 80 μrad/Nm, and compliance in roll 60 μrad/Nm. These imperfections will relate to some uncertainty contribution to the calibration measurements in the form of Abbé error.

### 2.2. Measurement Strategy

The act of sensor calibration is to experimentally determine factors such as the linearity and the amplification factor of the system. This can be achieved through the capture of sensor readings at discrete points across the operating range and comparison with a calibrated absolute scale. In the work reported here, three calibration approaches are investigated. Here, either a laser interferometer or one of two types of single material measure are used as a comparator to determine the relationship between sensor output and measured distance across the operating range of the sensor. In the case of the two single material measures, the first is a step-height constructed from tungsten carbide slip gauges (OPUS, U.K.) wrung onto an optically flat surface and the second is a glass coverslip (CMG 150, Excelitas Qioptiq). Both reference materials’ dimensions were calibrated by measurement on a Bruker NPFLEX. The relationship between the measurand value and the output of the sensor can be determined by following ISO 25178-600 [19], which mathematically represents this relationship as shown in Equation (1).

(1) $$\begin{array}{c} d_{in}=\alpha_{z}d_{C}+r, \end{array}$$

where $d_{in}$ is the measured quantity, $d_{C}$ is the actual quantity, and *r* represents the residuals due to random effects on the instruments’ indication that are normally distributed, and $\alpha_{z}$ is the amplification factor, relating instrument output to the measurand value and can be approximated via linear regression as shown in Equation (2).

(2) $$\begin{array}{c} \alpha_{z}=\frac{\sum_{i=1}^{n}d_{in,i}d_{C,i}}{\sum_{i=1}^{n}d_{C,i}^{2}}, \end{array}$$

where i=1,...,n are datapoints representing various distance offsets from the sensor. The following methodology sections outline how these datapoints are captured to derive this relationship.

#### 2.2.1. Method 1: Interferometer

Here, spectra from the spectrometer and positional data from a laser interferometer reference were simultaneously acquired whilst translating the mirror sample away from the fibre-tip as shown in Figure 1(1). The sample sat on top of a platform mounted to the vertical scanning stage with the retroreflector mounted directly below it. As a result, there would be some error contribution associated with the variation in straightness, flatness, pitch and yaw of the mechanical positioning stage platform amplified by the sample offset from the axis of travel. This positional error term presented itself in the form of the Abbé error, leading to a variable Cosine error in the calibration dataset calculated from Equation (3). This contribution could be minimised by using a common beam path for both the LCI and Renishaw XL-80 and by measuring in the axial plane of travel by using a vertical stage for varying the sample position. However, this greatly increases the complexity of the design and required the use of a vertical stage.

(3) $$\begin{array}{c} M=Zcos\theta \;,Z_{err}=Z\left(cos\theta -1\right), \end{array}$$

where *M* is the measured displacement, *Z* is the actual displacement along the axis of motion, θ is the angle between these two lengths and $Z_{err}$ is the resulting Cosine error between the two lengths.

The measurement procedure involved translations of 1 μm steps with 20 spectra and Renishaw XL-80 readings captured and averaged at each measurement location across a range of 2000 μm.

#### 2.2.2. Method 2: Step Height

This method looks at using a step height reference material as a cost effective alternative to a laser interferometer with the experimental setup shown in Figure 1(2). The step height was created by wringing two slip gauges of different thicknesses onto an optically flat surface next to each other and then measuring the resulting step height using a Bruker NPFLEX with the measured step height equaling 6.19 μm ± 0.03 μm. The sample was translated laterally across the LCI field of view in order to capture the step profile at a constant velocity of 0.1 mms${}^{-1}$. Due to the large measurement time involved here, the potential impact of thermal drift was assessed by taking measurements of a mirrored surface at a fixed offset from the sensor every 30 s for 7 h under the laboratory’s temperature controlled conditions with a maximum variation in measured offset of 0.15 μm detected. Hence, for the measurements performed here the impact of sensor drift was considered negligible on the calibration measurement. The calibration dataset was formed following an existing method [15]; this reduces the need for accurate positioning stages, creating an absolute scale through self comparison against a single calibrated material measure as shown in the following procedure:

1. Take a profile measurement from slip gauge 1 to slip gauge 2, where slip gauge 1 thickness > slip gauge 2 thickness;
2. Determine the distance offset of each slip gauge surface from the sensor;
3. Relocate the sensor to the lateral start position;
4. Move the sample away from the sensor until the sensor readout is the same as it was over slip gauge 2 in the previous line scan;
5. Repeat the process until the entire operational range is covered.

Limitations in the minimum stage incremental movement may limit the ability to align the new position to the previously read sensor frequency readout. To compensate for this variation in the absolute scale, a 2-point linear calibration can be carried out for each step and then this amplification factor can be used to determine the position of the next stage scanning start point. This correction can be calculated by Equation (4).

(4) $$\begin{array}{c} x_{i,correction}=\alpha_{i-1}x_{i}, \end{array}$$

where $x_{i,correction}$ is the corrected location, $\alpha_{i-1}$ is the compensation factor, and $x_{i}$ is the input location. A process outline [20] was followed during the creation of the reference material by wringing the slip gauges together with a settling period before measurement by the Bruker NPFLEX to calibrate the step height value. After measurement, the slip gauges were directly measured by the LCI system within a temperature controlled lab (20 °C) in order to minimise the change in step size due to thermal expansion. Thermal expansion can be determined through the use of Equation (5).

(5) $$\begin{array}{c} \Delta l=\alpha_{th}L\Delta T, \end{array}$$

where Δl is the change in sample length, $\alpha_{th}$ is the coefficient of linear thermal expansion (4.23 μm·m${}^{-1}\cdot$k${}^{-1}$), *L* is the sample nominal length and ΔT is the change in temperature. Gauge blocks of 1.001 mm and 1.007 mm thickness were used to create the reference material step height. Both laboratories were kept at standard environment conditions with a temperature of 20 °C. Hence, from Equation (5), the error contribution due to thermal expansion will be negligible. The nominal length of the reference material ($l_{n,s}$) is defined by Equation (6).

(6) $$\begin{array}{c} l_{n,s}=l_{n,1}-l_{n,2},\;\left(l_{n,1}>l_{n,2}\right), \end{array}$$

where $l_{n,1}$ and $l_{n,2}$ are the nominal lengths of each slip gauge block as defined in BS EN ISO 3650:1999 [21]. However, for a real-system of wrung gauges the gauge thicknesses are expected to vary from point to point on the unwrung measuring face of both gauge blocks. Therefore, the step height measurement achieved will vary depending on the location at which the step height measurement is taken across. From BS EN ISO 3650:1999 [21] the deviation from the nominal length is described as the limit deviation ($\pm t_{e}$). Hence, for every possible pair of points considered on each gauge face, the actual length ($L_{s}$) can be expressed by Equation (7).

(7) $$\begin{array}{c} l_{n,s}-2\cdot t_{e}\leq L_{s}\leq l_{n,s}+2\cdot t_{e}. \end{array}$$

The gauges used fall into the 0.5 mm $\leq l_{n}\leq 10$ mm category from Table 5 in BS EN ISO 3650:1999 [21], and as grade 0, $t_{e}=0.12$μm is given. Hence, from Equation (7) the step height should be within ±0.24 μm of the nominal step height $l_{n,s}$.

**Figure 2:**
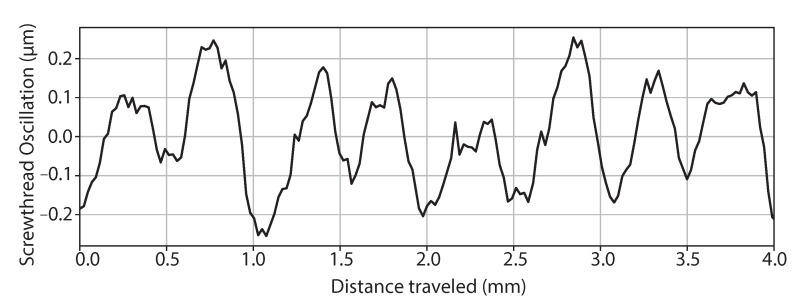
Screwthread pitch measured by in-house LCI system for x-axis motion.

Error contributions from stage movement are also present in the step-height scanning measurement. This can be minimised through the characterisation of the stages as described in the ISO 230 standard [22]. Figure 2 shows the variation from the fibre tip as a function of pitch deviation whilst translating the stage position from the measurement across an optical flat position atop the x-axis translation stage. Figure 2 shows that, for the x-axis region used, the stages exhibited a mean pitch of 490 μm and a mean oscillation of ±0.16 μm.

The measurement procedure involved laterally translating the step at 0.1 mms${}^{-1}$ past the sensing head with continuous capture of singular spectra across 4 mm. Due to the 125 Hz operating speed of the spectrometer, this gave a transverse datapoint density of 8 μm. After each lateral scan, the x-axis stage was homed and the sample translated away from the fibre tip by 6.19 μm until the sensor reading was approximately the same as when measuring the second slip gauge.

#### 2.2.3. Method 3: Glass Coverslip

To minimise the interaction of the sensor measurement and the mechanical system, and to significantly reduce the amount of time taken for calibration routines, the use of a transparent reference material is proposed as shown in Figure 1(3). This method builds upon the process outlined in method 2, utilising the same approach to creating an absolute scale based on the dimensions of a reference material. Here, an uncoated glass coverslip with a measured thickness of 152.26 μm ± 0.15 μm was used as a transparent reference material with a refractive index (RI) of 1.516 ± 0.003 as stated by the manufacturer. The tomographic potential of the LCI system was taken advantage of to capture signal frequencies relating to back reflection from both the top and bottom glass surfaces simultaneously. This removed the requirement for a time consuming scanning procedure across step heights allowing for the capture of multiple offset locations in one shot. Hence, this method combines many of the benefits from both method 1 and 2, yielding a simple, cost effective and robust mechanism of calibration. Compensation should be applied to the backreflected signal frequency from the bottom surface of the coverslip due to measurement through two mediums: the operating medium and the glass coverslip. Compensation can be achieved through finding the frequency offset relating to the coverslip thickness and then compensating for the change in RI between media.

The optical thickness, ΔD can be determined via multiplication of the physical sample thickness by the RI of the sample as shown in Equation (8). The sample RI and physical thickness, *t*, values can also be found through the comparison of two measurements; one where a reflective reference flat is undergoing measurement and then the second where the sample is placed in the measurement path in front of the reference flat with a spacer between the two [23,24,25].

(8) $$\begin{array}{c} \Delta D=n_{g}\cdot t, \end{array}$$

where $n_{g}$ represents the group RI averaged over the thickness of the sample. If the sample is then removed and the reference flat measured, the optical path length (OPL) relating to the sample would be replaced by an OPL related to the same linear distance travelling through air. This results in the axial position of the reference path shifting by Δω due to the change in OPL which can be calculated by Equation (9).

(9) $$\begin{array}{c} \Delta \omega =\left(n_{g}-n_{air}\right)\cdot t, \end{array}$$

where $n_{air}$ is the RI of air at standard conditions. Upon measuring both ΔD and Δω parameters, the sample thickness and group RI can be found simultaneously as displayed in Equation (10) and Equation (11) correspondingly.

(10) $$\begin{array}{c} t=\frac{\Delta D-\Delta \omega }{n_{air}}, \end{array}$$

(11) $$\begin{array}{c} n_{g}=\frac{\Delta D}{\Delta D-\Delta \omega }\cdot n_{air}. \end{array}$$

To correct for the OPL change due to the glass RI, a compensation term should be applied to the bottom identified signal frequency peak position. This is achieved via Equation (12).

(12) $$\begin{array}{c} x_{bottom\;shifted}=x_{top}+\frac{x_{bottom}-x_{top}}{n_{g}}, \end{array}$$

where $x_{bottom}$ and $x_{top}$ represent the signal offset from the bottom and top surfaces of the coverslip respectively and $x_{bottom\;shifted}$ is the actual location of the bottom surface, after compensating for the glasses, increased RI. The calibration measurement procedure involved translations of approximately 1 μm steps with 20 spectra captured and averaged at each measurement location across a range of 1680 μm.

## 3. Measurement Results

### 3.1. Calibration Results—Method 1: Reference Interferometer

The effect of operating with the sensor and sample submerged in various liquid media was investigated using the methodology outlined in Section 2.2.1 to determine the requirement for LCI sensor calibration across multiple operating domains.

**Figure 3:**
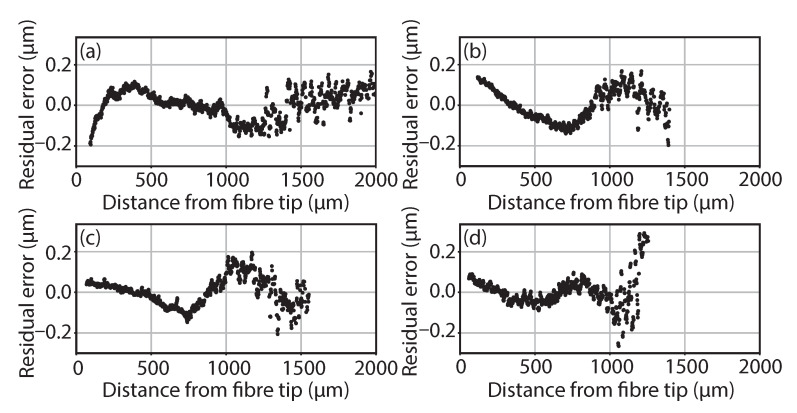
Residual errors from a 1st order polynomial fit to LCI captured readings versus Renishaw XL-80 readings across measured offset range, in (**a**) air, submerged sample/fibre tip in (**b**) paraffin oil, (**c**) water and (**d**) MWF.

The linearity of the sensor response, whilst operating in air, paraffin, water and MWF, can be seen in Figure 3 with their corresponding 2σ standard deviation (STD) shown in Table 1. Here, the residual error from a linear fit between the sensor measurement and the measured offset distance versus the distance from the fibre tip for each of the operating media is shown. The variance in measurement range between operating media is due to a reduction in the signal to noise ratio (SNR). This reduction is due to increased signal absorption in different liquids and from RI change leading to larger optical path differences (OPD) in the interferometer and, thus, signal fall-off [26]. The experimental setup used was able to operate over approximately 2000 μm in air, 1480 μm in paraffin, 1550 μm in water and 1200 μm in MWF.

Figure 3 shows an increasing deviation from the nominal at a larger OPL across all media; this is due to a reduction in the SNR leading to increased influence from random shot noise with some loss of datapoints. However, as can be seen, the deviation from the linear relationship across the measured range is still relatively small. This is also more pronounced when operating in media other than air, due to the increased signal dispersion and absorption effects. As the datapoint density remains high despite data loss, and due to the low deviation from a linear fit, the impact on the curve fitting analysis is considered to be negligible. As mentioned in Section 2.2.1, Abbé errors will also be present in the measurement results, leading to some non-linearity in the calibration. This error term will start as large for the Renishaw XL-80 measurement and small for the LCI measurement and then as the sample is translated away from the fibre tip, the error contribution will reduce for the Renishaw and increase for the LCI measurement.

As an approximately linear relationship can be observed across the operating range in the selected media from Figure 3, the acquired calibrated amplification factors can be applied between operating media so long as their respective change in RI is accounted for. Table 1 shows the estimated values for each medium’s RI from the literature, compared with the experimentally obtained values. Here, the RI is approximated by dividing the amplification factor of air by each medium’s acquired amplification factor; even a small level of deviation between these two results would have a large impact on the measurement result, especially at larger offsets if the literature defined results were used in place of the measured values. This is due to the relationship z=αx, where α is the amplification factor, *x* is the LCI sensor reading and *z* is the offset from the fibre tip.

### 3.2. Calibration Results—Method 2: Step-Height Reference Material

**Figure 4:**
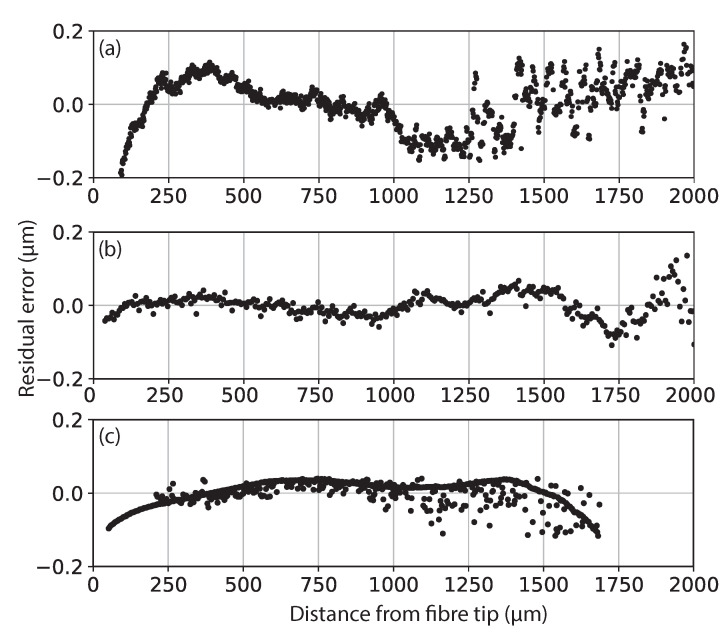
Residual errors from a 1st order polynomial fit to (**a**) LCI captured readings versus Renishaw XL-80 readings in air; (**b**) LCI captured readings versus step height readings in air; and (**c**) LCI captured readings versus glass coverslip thickness across measured offset range, in air.

Figure 4 shows a comparison between the three stated calibration methods in Section 2.2; the residual error from a linear fit between sensor reading and the actual z-translation measured in air. The residual error between the reference result and the calibrated input for a linear fit to the step height calibration data is shown in Figure 4b with a calculated 2σ STD of 0.026 μm. This shows a significant reduction in residual error versus the dataset captured with comparison to the laser interferometer in Figure 4a. This is thought to be mainly attributable to the reduction in Abbé errors due to the different experimental setups. The scanning procedure also allows for the capture of a greater number of datapoints with the determination of each slip gauge face being calculated by taking the mean value; this acts as an additional signal filter reducing the impact of random shot noise on the signal peak at a larger OPD.

### 3.3. Calibration Results—Method 3: Coverslip Reference Material

Figure 4c shows the residual for a linear fit across approximately 1680 μm; measurement beyond this point yielded a backscattered signal from the bottom side of the coverslip below the system’s noise floor. The reduction in operation range is due to a portion of the signal being backreflected from the top surface, hence only a fraction of the signal will reach the bottom surface of the coverslip; this may be improved by coating the top and bottom surface with different reflective materials such that a greater proportion of the signal will be backreflected, increasing detected signal strength. Due to the thickness of the coverslip, only several datapoints can be captured if the stage is translated by the thickness of the sample after each measurement. Hence, here multiple points are captured within the thickness of the coverslip. This is achieved by translating the sample in approximately 1 μm steps and using a 2 point linear fit from the known relationship between the top and bottom sample surface’s signal response and spatial distance to create the absolute y-axis similar to the previously described method in Section 2.2.2.

This method produced a 2σ STD of ±0.077 μm from a linear fit across the measured range, which again demonstrates a reduction in residual error from the laser interferometer measurement as a result of the potential removal of mechanical errors from the measurement. This mode of operation should be the most robust with the least impact from potential Abbé and Cosine errors on the dataset and, due to the single point capture, the calibration process is much faster than the step height scanning process (approximately 21 s versus 3.6 h for the same translation speed and step resolution). However, it can still be seen that the step height measurement was able to provide a more linear response across the full range of operation. This may be due to errors introduced from the 2 point position fitting procedure and due to the scanning operation acting as a signal filter averaging out deviations in the step height measurement result, leading to a lower overall residual error.

### 3.4. Method Comparison

Although the step heights appear to provide a better set of results there are advantages and drawbacks to all the methods presented as highlighted in Table 2.

### 3.5. Residual Error and Dataset Sparsity

The Renishaw vs. LCI datasets used to generate Figure 3 were also used here to quantify dataset sparsity’s impact on calibration curve quality across the tested media. Here, the datasets were sampled with a frequency of $\left[2...N\right]$, where *N* is the maximum number of points in the dataset. In order to generalise the impact of sampling number, datapoint selection was carried out randomly using Python’s random.sample() library function. This process was then repeated for 5000 iterations to obtain the mean trend of residual error STD versus the number of datapoints. The process outline is shown via the following steps:

1. Set the number of points to sample;
2. Get the index of the points using Python’s random.sample() library function;
3. Calculate a linear fit using the points acquired;
4. Calculate the residual error using the linear fit coefficients for the entire dataset;
5. Calculate the 2σ STD of the residual error;
6. Once all of the set iterations have been completed, calculate the average 2σ STD for each dataset frequency.

**Figure 5:**
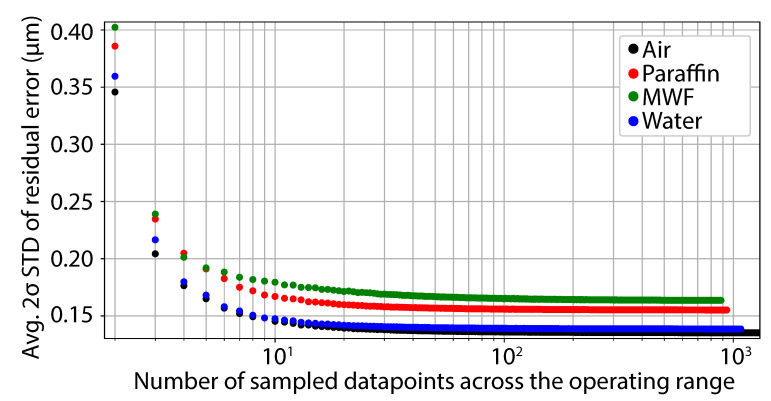
Dataset sparsity impact on calibration curve residual error in (black) air, submerged sample/fibre tip in (red) paraffin oil, (green) MWF and (blue) water.

The average 2σ STD of the obtained residual errors for each sampling frequency is shown in Figure 5. Due to the LCI sensor’s response being approximately linear in nature, as shown in Figure 3 and Figure 4, the impact of data sparsity is largely attributable to noise terms in the readings. Hence, the reduction in residual error is brought about by increasing the fitted datapoints to compensate for this random noise term. This behaviour is shown in Figure 5, where a convergence pattern in the residual error STD can be seen with increasing datapoint density. It should be noted that, in accordance with Figure 3, the random noise term increases at a larger OPD and where there is a lower SNR. Hence, sampling datapoints at a lower OPD would also relate to a better representation of the signal with a smaller number of required datapoints. A logarithmic x-axis is used to present the dataset, which covers 3 orders of magnitude.

## 4. Conclusions

Three universal methods for calibrating an LCI system have been shown: a comparison of a laser interferometer versus two types of singular reference materials used to build a calibration profile across the entire range of operation. The two reference materials require different measurement procedures due to their structure; the first included performing a scanning measurement across a step height and the second using a single capture of a coverslip thickness to provide two point calibration. The comparison between step height, coverslip and interferometer calibration demonstrates that the use of step heights or coverslips are valid alternatives and even offer some benefits over the interferometer in terms of simplicity, robustness and cost. The impact of operating media on the calibration results has been investigated, showing an approximately linear response in the selected media. Hence, a singular calibration can be acquired in one medium and transferred for use in other media so long as the sensor’s change in sensitivity due to variance in RI is accounted for. Finally, the requirement for high resolution calibration across the region of operation was investigated for on-machine metrology, where limited downtime is of priority; it was seen that, due to the linearity of the system, the main contributor was a random noise term, hence, increasing datapoints, especially at a smaller OPD, will allow for an accurate determination of the system’s amplification factor.

## Figures and Tables

**Table 1:** Comparison of residual error from 1st-order polynomial fit across specified operating media, with RI values taken from measured amplification factors and from the literature.

Media	RI ${}^{†}$	RI ${}^{‡}$	STD 2σ (μm)
Air	1.00	1.00	±0.135
Water	1.34	1.33 [27]	±0.144
Paraffin	1.44	1.47 [28]	±0.155
MWF	1.35	1.48 [29]	±0.159

† experimentally measured RI, ‡ RI values approximated from literature.

**Table 2:** Advantages and disadvantages of each calibration approach explored.

	Interferometer	Step Heights	Glass Coverslip
**Positives**	Fast—21 s	Inexpensive	Fast—21 s
	Variable resolution	Simple to use	Inexpensive
	Versatile	Increased result stability	Least interaction with mechanical system
		Variable step heights	Simple to use
			Can stack for multi-depth measurements
			Robust
**Negatives**	Expensive equipment	Very slow—3.6 h	Fixed resolution
	Increased impact from Abbé errors & Cosine errors	Thickness variation produces calibration uncertainty	Thickness variation produces calibration uncertainty
	System complexity	Prone to wringing errors	Resolution limited to coherence length
	Difficult to setup	Fixed resolution	Fixed resolution
		Influence from mechanical system	Weak signal response at high OPD

## Data Availability

The data presented in this study are openly available in FigShare at https://doi.org/10.6084/m9.figshare.15060477.v1 (accessed on 7 June 2021).
